# Analysis of Surface Geometry Changes after Hybrid Milling and Burnishing by Ceramic Ball

**DOI:** 10.3390/ma12071179

**Published:** 2019-04-11

**Authors:** Daniel Grochała, Stefan Berczyński, Zenon Grządziel

**Affiliations:** 1 Faculty of Mechanical Engineering and Mechatronics, West Pomeranian University of Technology, 19 Piastów Ave., 70-310 Szczecin, Poland; daniel.grochala@zut.edu.pl (D.G.); stefan.berczynski@zut.edu.pl (S.B.); 2 Faculty of Marine Engineering, Maritime University of Szczecin, 1-2 Wały Chrobrego St., 70-500 Szczecin, Poland

**Keywords:** surface geometry, milling, burnishing by ceramic ball, hybrid machining, FEM modeling

## Abstract

The production of modern machines requires parts with much greater geometric accuracy and surface geometry (SG) precision than several years ago. These requirements are met by so-called hybrid technologies that must simultaneously be inexpensive to implement. The integration of treatment procedures (usually in one operation) is geared towards achieving a synergistic effect. Combining different treatments from various technologies produces synergy, i.e., benefits greater than the optimization of each individual process done separately. This paper presents experimental results and numerical experiment data on surface plastic deformation. The hybrid technology used in the study was a combination of milling and finishing with plastic burnishing using a ceramic ball. These processes were integrated on a multi-axis CNC machining center. The plastic deformations of real surfaces were determined in simulations. The paper also discusses the structure of the model and how to use it to conduct a finite element method (FEM) computer simulation. The aim of the study was to determine how to use the potential developed model of hybrid treatment to predict the surface performance expressed by the amplitude, volume, and functional parameters of the surface geometry, with the EN-ISO 25178-2 profile.

## 1. Introduction

The current machine part technology is becoming more integrated (one machine, a few tools, short production time and cost reduction). Research papers and industrial practice focus on the combination of machining types [1,2,3,4,5,6,7,8,9], including turning [10], milling [11,12,13,14,15], drilling, and threading, using machining centers with forming processes like burnishing [1,2,3,4,5,6,7,8,9,10,11,12,13,14,15,16,17,18]. The main aim of the new technology is to meet the smoothness and strength requirements of the surfaces of the part [1,2,3,4,5,6,7,8,9,10,11,12,13,14,15,16,17,18,19,20,21]. The optimization of the technological parameters of turning [10] or milling [5,6] makes it possible to obtain a satisfactory surface geometry (with low roughness and a useful Abbott–Firestone curve). Given the predefined accuracy, the efficiency and production economics are the main concerns for the technologist. The practically unlimited freedom to control the tooth path trajectory on multi-axis milling machines enables the technologist to use a range of smart tools to obtain the required properties for useful surface shapes [2,3,4,5,6,18].

In addition to the stress responsible for the fatigue strength, the performance properties of the surface [6] also include a new range of additional requirements defined by constructors and R&D specialists. A more common new requirement is a given level of surface isotropy [7,8]. It is beneficial to prepare a surface with an isotropic geometric structure when the operation conditions have not been completely defined. An isotropic surface results in the even wear of components during their use. Given a low roughness for the surface geometry, an isotropic surface provides a uniform and high surface reflectance in all the regions, which is particularly important in modern industrial design [7,8,19]. The surface texture obtained with hybrid production techniques depends to a large degree on kinematic–geometric conditions and the technological parameters defined by the technologist. The surface of a ready product often bears traces of the treatment applied prior to surface finishing [7,9,10,11,12]. Working through the remains of the last treatment poses a significant limitation to synergy in hybrid technological processes [7].

In the production of expensive and complex tools, including molds, masters, and dies, there is no room to search for the optimum treatment parameters that would produce the synergy effect. This paper presents a way to develop a finite element method (FEM) model and research methodology for the assessment of the height parameters of a surface geometry. The model data were validated with experimental data. An analysis covered selected parameters of the surface geometry.

## 2. Experimental Research

Complex spatial surfaces are commonly machined with ball nose end mills and torus heads. Milling cutters leave traces related to the feed rate, interval, and diameter of the cutting insert. The roughness resulting from the feed per revolution is usually negligibly small. In such cases, the standard procedure is to burnish perpendicular to the direction of the crossfeed cutting after milling [4,12,13,14,15,16]. The burnishing itself is accompanied by phenomena occurring in the contact zone of the burnishing tool. The effect of burnishing to a large degree depends on the hardness of the material and roughness resulting from the previous operations. The basic technological parameters of burnishing include the burnishing force, feed, and number of burnishing tool passes. The surface condition is the least affected by the burnishing velocity.

**Figure 1:**
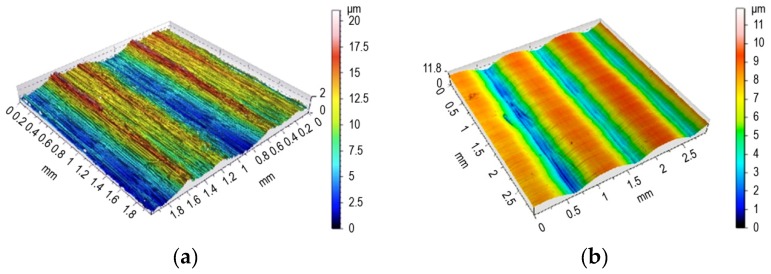
(**a**) Surface obtained after milling; (**b**) Surface after milling and burnishing.

An experiment was planned in line with the literature guidelines [9,10,11,12,13,14,15,16] and based on our own experience in conducting experimental research [5,6,7,8]. First, 100 × 100 × 20 mm samples made of 42CrMo4 steel and thermally improved to 35 ± 2 HRC were milled using a WNT R1000G.42.6.M16.IK torus (Ceratizit Group, Poland) with six inserts to a diameter of *d_p_* = 10 mm (RD.X1003 MOT–WTN1205). The treatment was conducted on a DMG DMU 60MONOBLOCK machining (Deckel Maho Pfronten GmbH, Pfronten, Germany) center, with a spindle angle of 15°. The milling speed was *v_c_* = 1000 mm/min, with a feed rate *f_z_* = 0.1 mm and a cross feed rate *f_wm_* = 0.5 mm. An approximately 11 mm wide sample was left unburnished to measure the roughness obtained in the forming milling (Figure 1a).

Measurements of the surface geometry (SG) were made using an AltiSurf A520 multisensor (Altimet, Thonon Les Bains, Frence). It was fitted with a chromatic confocal CL1 sensor, with a range of up to 130 µm and a resolution of 8 nm on the Z optical axis. Measurements were conducted on 2 × 2 mm areas. A scanning resolution of 2 µm along the X and Y axes was experimentally selected. As a result, a cloud of 1 million points was obtained. The mapping of this point cloud was conducted using an AltiMap PREMIUM 6.2 (Digitalsurf, Besancon, France), and then 3D SG parameters were determined:

- for each of the measured areas, a threshold value of 0.01–99.9% was determined to delete unreliable surface point data (deleted points were set up as unmeasured values);
- then, the surface was leveled (with mean area using the method of least squares);
- finally, selected stereometric roughness parameters were determined in compliance with EN-ISO 25178.

Selected SG parameters after milling are listed in Table 1. The cloud of points for a real surface prepared in this way was exported using the TXT file standard. A recorded image of the milled surface was used for a numerical analysis conducted with the software used for modeling the plastic deformation with the FEM.

The experimental research on burnishing was conducted on a 3-axis MIKRON VCE 500 machining center (Haas Automation INC, Oxnard, USA). A prototype hydrostatic tool with a bellows actuator (West Pomeranian University of Technology Szczecin, Szczecin, Poland) (Patent PL 220528 B1 issued on Nov. 30, 2015) with a ZrO_2_ ceramic tip with a diameter of *d_b_* = 10 mm was used. The burnishing speed *v_b_* was 8000 mm/min, and cross feed *f_wb_* was 0.12 mm. Burnishing was conducted with a force *F_b_* = 500 N. The surface after burnishing is shown in Figure 1b.

After burnishing, selected 3D SG parameters were again determined (Table 1).

## 3. Computer Model of Burnishing Process

**Figure 2:**
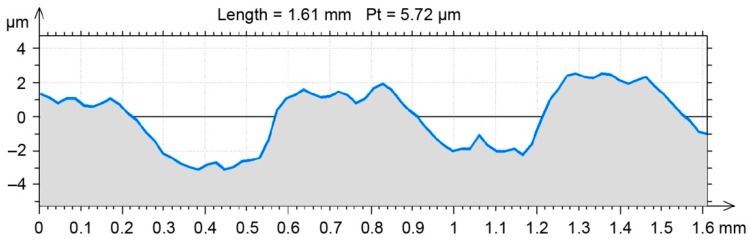
Randomly selected profile used to model burnishing.

A randomly selected milled profile from an earlier generated point cloud for a real surface was used to analyze the SG changes following hybrid treatment in the FEM environment (Figure 2).

**Figure 3:**
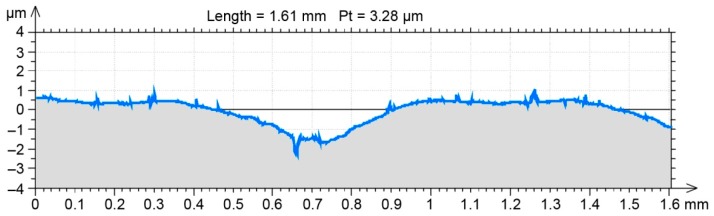
Randomly selected benchmark profile obtained in burnishing experimental research.

A randomly selected milled profile was set as a benchmark for the FEM environment results (Figure 3). After burnishing, the profile was obtained in the same way as the profile of the milled surface. Selected 2D SG parameters (Table 2) were determined in compliance with the EN-ISO 4287 standard for both profiles (Figure 2 and Figure 3).

Milling with ball nose cutters leaves marks, with the feed per revolution and ball nose cutter diameter as the dominant factors in the roughness. Burnishing smooths the marks.

**Figure 4:**
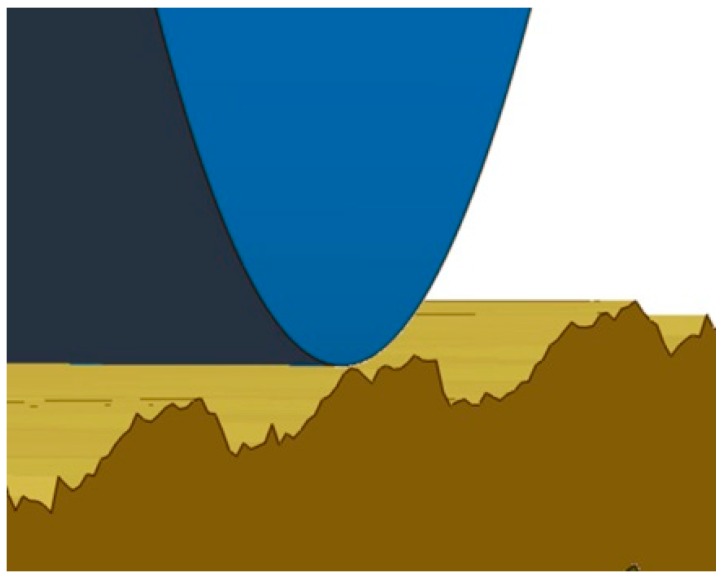
Model of surface burnishing developed in a finite element model (FEM) environment.

An earlier numerical simulation [6] demonstrated in a sensitivity analysis that a mesh gap size below 50 µm did not significantly affect the plastic deformation and residual stress of milled and burnished surfaces. Therefore, bearing in mind the hardware limitations of the physical model developed with Nastran FX, a mesh gap size of 20 µm was used for an individual finite element. Roughness shape changes were observed in a selected milled surface profile in the physical model. The changes visible in Figure 4 are a result of changing the measurement mesh (2 × 2 µm) into a (20 × 20 µm) FEM mesh.

**Figure 5:**
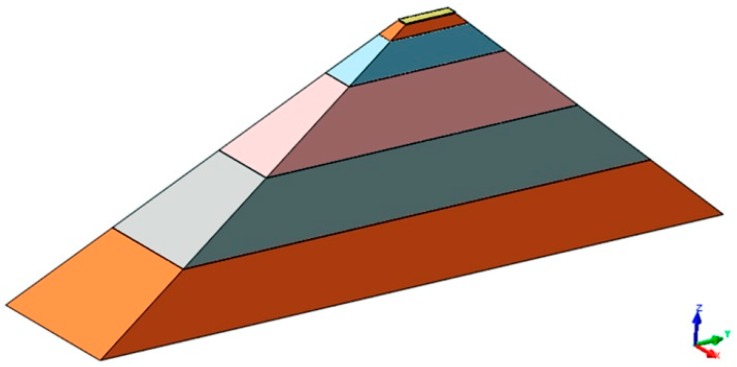
Sample prepared for Nastran FX simulations.

The samples used in the experiments were 0.4 mm wide and 2 mm long. The modeled physical object was similar to a pyramid with the top cut off. It was 10 mm in height, with a base that was 10 mm wide and 25 mm long (Figure 5).

The material properties of the thermally improved milled sample were taken into consideration. Nonlinearity was attributed to the sample material to account for the residual stress resulting from prior milling. The characteristics of the material used in the physical model were determined in tensile testing (Table 3) [6]. 

**Figure 6:**
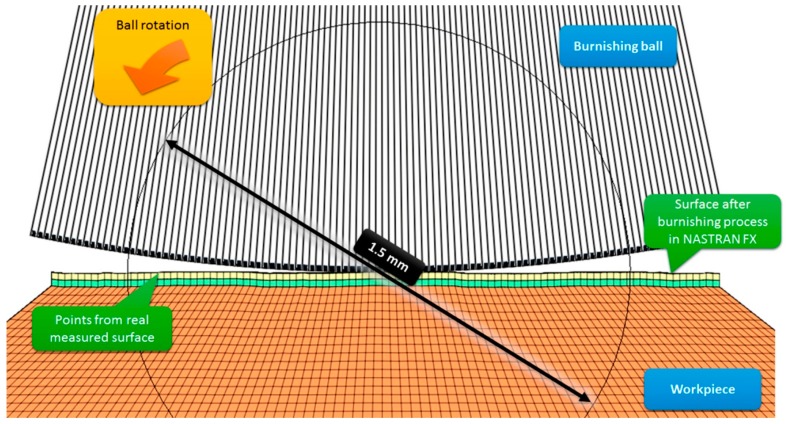
Sample model at contact point when ball is rolled back on real surface.

Young’s modulus of X42CrMo4 was determined experimentally; it was E = 210 GPa. Poisson’s number for the model was ν = 0.28 [6]. In the burnishing model, a ball was pressed into the sample surface with a force of 500 N. The ball was then rolled back perpendicularly to marks left by the milling cutter over 1.5 mm (Figure 6).

The model of a sample shaped like a pyramid with a cut off top consisted of 470,400 elements connected by 247,222 nodes. The base of the sample was fixed so that displacement was possible only in the model’s plane of symmetry. A total of 2100 rigid shell elements connected by 2227 nodes were used to model the ceramic tip of the burnishing tool. The mechanical properties of ZrO_2_ were taken from research conducted previously: Young’s modulus *E* = 220 GPa and Poisson’s number *ν* = 0.3.

The modeling of the burnishing process (Figure 6) comprised the following steps:

- modeling of how the ball is pressed into the sample surface with a burnishing force of 500 N;
- then, the burnishing ball is rolled back over the surface over a 1.5 mm section;
- the burnishing force acting on the ball is reduced.

When the ball is rolled back, it rotates by approximately 18°. It took 48 h for Nastran FX to calculate the data for one numerical experiment. The results included the plastic deformation of the total model and coexisting residual stress.

## 4. Experimental Research Results

**Figure 7:**
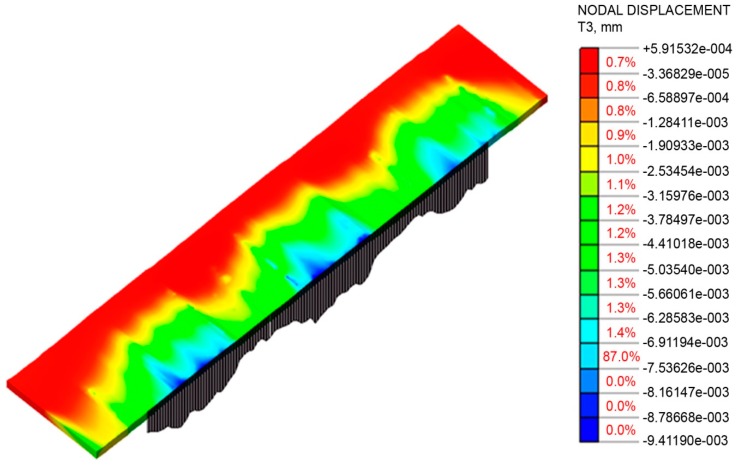
Plastic deformation of sample after one pass of the burnishing tool with a force of 500 N.

Because of hardware limitations and the high requirements for computing power in numerical research, the selected segment of a real milled surface was only 1.5 mm long. Therefore, only unfiltered profile P-parameters were calculated in compliance with EN-ISO 4287 (over one measured section) out of the vertical displacement data recorded in the direction of the burnishing ball rolling back (Figure 7). The value of the Pa-parameter can be easily interpreted by analogy with the Ra-parameter (the arithmetic mean deviation of the unfiltered profile points away from the midline).

**Figure 8:**
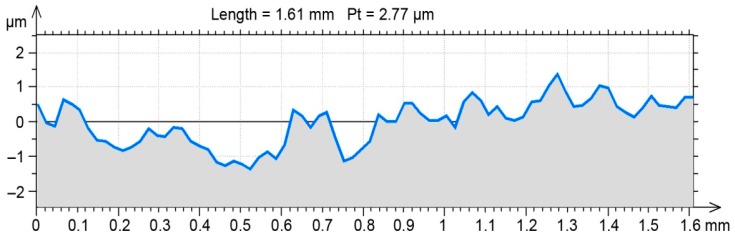
Milled surface profile after the simulation of a burnishing process with Nastran FX.

To determine the selected 2D SG parameters (Table 4) after the burnishing simulation, the set of points was exported again (Figure 8), and the SG parameters were calculated using AltiMapa 6.2.

The value of Pa = 0.55 was obtained in the burnishing simulation for a selected milled profile from the initial roughness of Pa = 1.56. The differences in parameter Pa were convergent to the average parameter values obtained in experimental research (Table 2 and Table 4). The experimental and simulated Pa-parameters differed by less than 5% of their values (Table 4).

**Figure 9:**
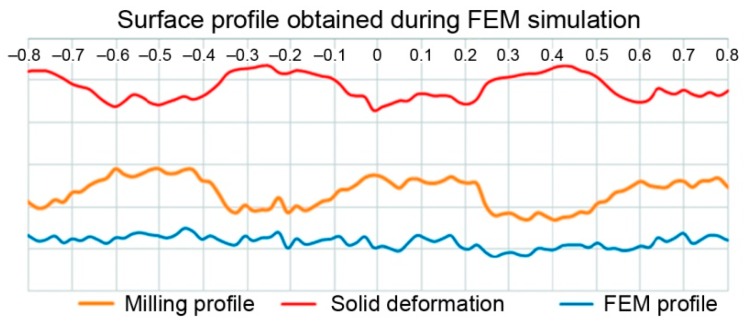
Graphs show surface profile after milling, solid deformation in investigated profile and final profile after burnishing with a force of 500 N.

The profile changes in the workpiece surface during the numerical research are shown in Figure 9. The biggest deformations were observed close to “sharp” summits. Summit deformations caused by the force exerted by the tool in the normal and tangential directions to the surface resulted in valleys being evenly filled in the whole area. In this particular burnishing case, lowering the surface peaks rather than elevating the valleys resulted in the plastic “filling” of the profile. As can be seen, there was a larger difference in the values of the Pp-parameters obtained after burnishing compared to the lowering of the Pv value of the burnished surface in relation to the original value obtained after milling. The trend observed in the numerical research was also confirmed in the experiment.

**Figure 10:**
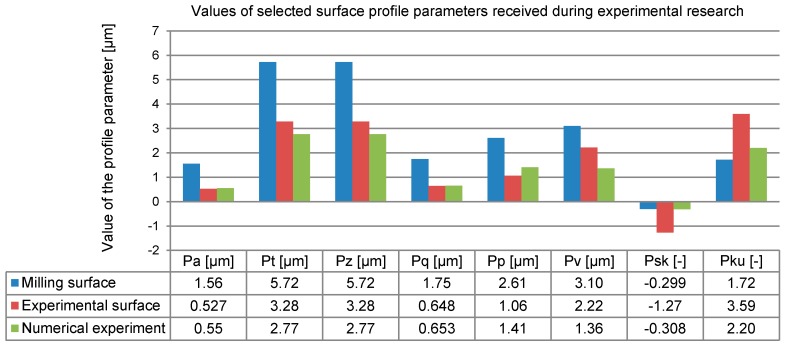
Changes in 2D SG parameters in the unfiltered profile.

**Figure 11:**
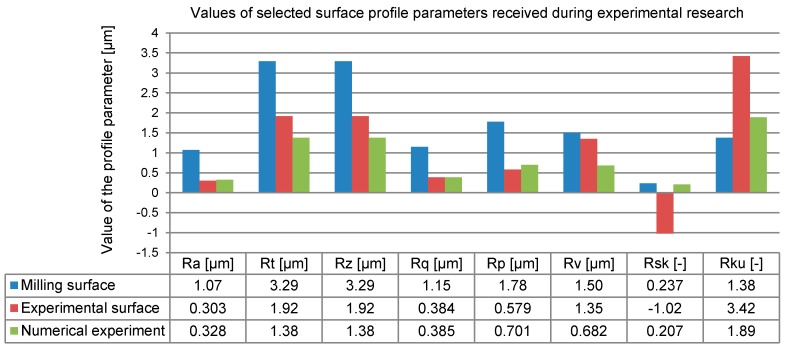
Changes in 2D SG parameters in roughness profile (profile filtered in compliance with EN-ISO 4287).

Small differences (of less than 10%) between the experimental and numerical results were observed only for the parameters most commonly used in engineering: Pa and Pq (Figure 10). Similarly, a small difference was observed in the filtered profile for roughness parameter Ra (Figure 11). The values of the 2D SG parameters depended on all the points in a set generated by a profile whose changeability was constant (for Pa) or the root mean square (for Pq) relative to a determined value of the arithmetic mean.

The goodness of fit between the experimental model and experimental results for unfiltered profile amplitude parameters Pt and Pz was worse. In this case, the coordinates of individual points were crucial in terms of their values. The extreme displacement of individual points on a surface decreased when the FEM mesh gap size increased (resolution of the FEM model decreased). Similarly, great differences were observed for the 2D SG parameters that defined the profile ordinate distribution, including the Psk skewness and Pku kurtosis. The observed asymmetry of the unfiltered profile point distribution and concentration of points around the average were greater in the experimental research. This was due to the very high resolution of the profilometer used in the study. The observed direction of changes in the surface character following the use of the hybrid technology was consistent. Some similarity can also be seen in the description of the surface changes expressed with the roughness amplitude parameters (Figure 11).

As before, an excellent goodness of fit was obtained for the Ra and Rq-parameters (differences smaller than 10%). The numerical research could be used to satisfactorily predict the mechanism and magnitude of the deformation of the roughness summits after milling (the Rp differences were around 21%). Unfortunately, the characteristic of the valley filling in the roughness profile defined by the Rv parameter yielded a difference of almost 10%. Just like before, the reasons for such a great magnitude of errors can be found in the FEM model discretization, which was ten times smaller than that used in the experimental research.

The point distributions of the burnished profile were characterized by different directions of asymmetry (parameter Rsk). The high resolution of the measuring equipment also played a role in this respect. High resolution could be used to better scan and map the narrow and deep valleys of the real burnished surface.

The developed FEM model could satisfactorily determine the Rmr and Rdc parameters of the core ratio of the material. Values based on the profile ordinate distribution given by the Abbott–Firestone curve are often used by technologists to define the required tribological properties of a product surface. To determine the material core parameters, the extreme points at a profile’s summits and valleys are rejected.

## 5. Summary and Conclusions

The following conclusions can be drawn based on a comparison of the numerical research data and empirical experiment data.

Because of hardware and software limitations, only one pass of burnishing was modeled in the initial research. This is why the surface residual deformation results from the numerical experiment and experimental research were somewhat divergent. The origin of the observed differences lay in the propagation of the residual deformation and stress outside the direct contact zone between the workpiece and burnishing tool. This practically means that each successive burnishing pass occurred on a surface that had already been burnished.

In its present state, the developed numerical burnishing model is a good prognostic tool for predicting the condition of a surface layer. It is particularly useful for residual stress forms and values, which are difficult to measure.

In the numerical research, we assumed the real condition of a milled surface scanned with 0.002 mm steps. The first simulation attempts showed that 0.02 mm steps would be good enough for computer modeling. This simplification did not significantly affect the surface height parameters obtained in the study.

The differences in the parameters typically used to define SG were in most cases below 20%. The assumed simplification made it possible to reduce the computing time and did not significantly affect the ability to predict the SG conditions.

The developed model of the treatment process could also be used to predict the fundamental tribological properties of a surface. The profile point distributions generated with the model were consistent with the experimental data.

In special cases, amplitude parameters, defined by individual points with extreme height values, could not yet be estimated. However, our research is ongoing. First, we plan to increase the resolution for the mesh discretization in the FEM model. Then, we want to introduce additional burnishing passes so that stereometric SG parameters (defined by EN-ISO 25178) can be determined.

Only 3D simulations will provide a reliable tool for the optimization of technological parameters in a hybrid treatment that includes milling and burnishing. A 3D model with improved resolution will enable the prediction of other functional surface properties such as isotropy, wetting, or the ability to maintain lubricants and lacquer coatings.

## Figures and Tables

**Table 1:** Selected 3D surface geometry (SG) parameters recorded in experiments.

SG Parameters in Compliance with EN-ISO 25178				After Milling	After Burnishing
Parameter Name	Parameter Description	Context	Unit		
Sa	Surface height arithmetic mean		µm	1.89	1.70
Sz	Maximum surface height		µm	14.40	11.90
Sq	Root mean squared surface height		µm	2.33	1.94
Ssk	Surface asymmetry			0.42	−0.46
Sku	Surface kurtosis			2.67	1.96
Sp	Surface peak maximum height		µm	9.05	5.45
Sv	Surface valley maximum height		µm	5.38	6.49
**Functional parameters (stratified surfaces)**					
Sk	Core roughness depth	Gaussian filter, 0.08 mm	µm	1.03	0.15
Spk	Reduced summit height	Gaussian filter, 0.08 mm	µm	0.53	0.13
Svk	Reduced valley depth	Gaussian filter, 0.08 mm	µm	0.57	0.18

**Table 2:** Selected 2D SG parameters recorded in experiments.

SG Parameters in Compliance with EN-ISO 4287				After Milling	After Burnishing
Parameter Name	Parameter Description	Context	Unit		
**Amplitude parameters—fundamental profile**					
Pa	Arithmetic mean deviation of primary profile		µm	1.56	0.527
Pt	Total height of primary profile		µm	5.72	3.28
Pz	Maximum height of primary profile		µm	5.72	3.28
Pq	Root mean squared deviation of primary profile		µm	1.75	0.65
Pp	Maximum peak height of primary profile		µm	2.61	1.06
Pv	Maximum valley depth of primary profile		µm	3.10	2.22
Psk	Primary profile asymmetry			−0.29	−1.27
Pku	Primary profile kurtosis			1.72	3.59
**Amplitude parameters—roughness profile**					
Ra	Arithmetic mean deviation of roughness profile	Gaussian filter, 0.8 mm	µm	1.07	0.30
Rt	Total height of roughness profile	Gaussian filter, 0.8 mm	µm	3.29	1.92
Rz	Maximum height of roughness profile	Gaussian filter, 0.8 mm	µm	3.29	1.92
Rq	Root mean square deviation of roughness profile	Gaussian filter, 0.8 mm	µm	1.15	0.38
Rp	Maximum peak height of roughness profile	Gaussian filter, 0.8 mm	µm	1.78	0.58
Rv	Maximum valley depth of roughness profile	Gaussian filter, 0.8 mm	µm	1.50	1.35
Rsk	Roughness profile asymmetry	Gaussian filter, 0.8 mm		0.24	−1.02
Rku	Roughness profile kurtosis	Gaussian filter, 0.8 mm		1.38	3.42
**Material balance parameters—roughness profile**					
Rmr	Relative material balance of roughness profile	c = 1 µm under the highest peak, Gaussian filter, 0.8 mm	%	35.90	81.50
Rdc	Height difference of roughness profile parts	p = 20%, q = 80%, Gaussian filter, 0.8 mm	µm	2.36	0.66

**Table 3:** Properties of the steel X42CrMo4, used in experiments (35 HRC) [6].

Parameter	Symbol	Unit	Catalogue Data for 20–24 HRC	Average Value in Tensile Tests 35 ± 1 HRC
Longitudinal modulus of elasticity	EX	GPa	210	210.2
Poisson’s ratio	NUXY	-	0.28	0.28
Longitudinal modulus of elasticity	GXY	N/m2	7.9 × 10^10^	-
Tensile strength	SIGXT	GPa	1.000	1.046
Yield strength	SIGYLD	MPa	750	840
Coefficient of thermal expansion	ALPX	/K	1.1 × 10^−5^	-
Elongation	A	%	14.7	10.86

**Table 4:** Selected roughness parameters recorded in the study.

EN-ISO 4287	Amplitude Parameters—Primary Profile				
	Unit	Milled Surface	Empirical Experiment	Numerical Experiment	Difference [%]
Pa	µm	1.56	0.52	0.55	4.4
Pt	µm	5.72	3.28	2.77	18.4
Pz	µm	5.72	3.28	2.77	18.4
Pq	µm	1.75	0.65	0.65	0.8
Pp	µm	2.61	1.06	1.41	33.0
Pv	µm	3.10	2.22	1.36	63.2
**Amplitude parameters—roughness profile**					
Ra	µm	1.07	0.303	0.33	8.3
Rt	µm	3.29	1.92	1.38	39.1
Rz	µm	3.29	1.92	1.38	39.1
Rq	µm	1.15	0.39	0.39	0.3
Rp	µm	1.78	0.58	0.70	21.1
Rv	µm	1.50	1.35	0.68	97.9
**Material balance parameters—roughness profile**					
Rmr	%	35.90	81.5	72.50	12.4
Rdc	µm	2.36	0.66	0.82	25.1
